# 

**Published:** 1913-05-17

**Authors:** 


### Perforated Duodenal Ulcer.

The revolution in surgical knowledge and teaching concerning the frequency, symptoms, diagnosis, and treatment of duodenal ulcer has been very largely the work of Sir Berkeley Moynihan, of Leeds, and many surgeons, guided by his teaching, have been enabled to improve treatment and to save life. This point is illustrated by Mr. J. W. Struthers in a paper which has been published in the *Edinburgh Medical Journal*, summarising the results of twenty-seven cases of perforated duodenal ulcer in his own practice. Only seven out of the twenty-seven patients died, and two of these were so bad when first seen that it was impossible to operate on them. The operation mortality works out therefore at no more than 20 per cent. Mr. Struthers emphasises especially the following points: (1) Duodenal ulcer may be present and lead to perforation without giving rise to characteristic symptoms before perforation; (2) There are no symptoms which may justly be described as premonitory of perforation; (3) Difficulty in diagnosis may arise owing to pain being referred to the appendix region, or to abatement of symptoms where leakage is limited by early adhesions or by œdematous swelling of the intestinal wall in cases of very minute perforation; (4) Catgut may safely be used to close and infold the perforated ulcer, and is preferable to silk for this purpose; (5) Gastro-enterostomy should be done at the operation for closing a perforated duodenal ulcer when the patient's condition will permit of it.

### Obituary.

Mr. Augustus Phillips Hills, M.R.C.S., has died at Battersea, where he was in practice for thirty years. He studied at Guy's, and took his M.R.C.S. in 1880. He had formerly held the post of vice-president of the Incorporated Medical Practitioners' Association. His family since 1736 were established at Arundel, and were surgeons to the Dukes of Norfolk.

Mr. Peter Herbert Metcalfe, M.R.C.S., has died suddenly at Fanning Island, North Pacific. As Government medical officer at Norfolk Island in the 'seventies, he became attached as medical officer to the Melanesian Mission, which had its headquarters there, and retained this post till 1910. This work was, perhaps, the main interest of his life.

### THE COMPLETE PAD OR DRAW-SHEET SUBSTITUTE.

In the reference made in last week's issue to the "Complete Pad" or draw-sheet substitute, exhibited by Dr. G. Vickerman Hewland at a recent meeting of the Hastings Division of the B.M.A., it should be understood that the patient is intended to lie on the surface of the pad opposite to the slot; the layer of absorbent wool should therefore be inserted before the mackintosh.

### Buildings Contemplated.

Lymington.—The foundation-stone of a new cottage hospital has been formally laid.

Millom and Booth.—It has been decided to add four additional wards, with nurses' quarters and improved sanitary provisions, to the Millom and Booth Joint Isolation Hospital. The cost is estimated at £1,000.

Monyhull.—The Birmingham Guardians have obtained the sanction of the Local Government Board to an expenditure of £88,400 upon the erection of buildings and the provision of fittings at Monyhull Colony. Sanction to the loan for furnishing has been deferred.

Newcastle, New South Wales.—A site has been selected for a new hospital.

Ripon.—It is proposed to improve the workhouse hospital at a cost of £2,000.

Petersfield.—The Local Government Board has held an inquiry into an application by the Southampton County Council for permission to borrow £2,700 for extending the isolation hospital of the Petersfield Hospital Committee. The plans are by Mr. H. T. Keates, of Petersfield.

Taylorton.—It has been decided by the Stirlingshire Central District Committee to build a smallpox hospital at Taylorton, near Stirling, at an estimated cost of £1,550. The hospital will consist of a ward block of eight beds and an administrative block and offices.

Thornhill.—Itchen Urban Council has resolved to apply for borrowing powers for the purchase of a site for an isolation hospital.

Tiverton.—The Rural Council has agreed to the proposal of the County Council to erect a sanatorium on a site near the isolation hospital at a cost of about £2,000.

Tooting Bec, S.W.—The Metropolitan Asylums Board have decided, subject to the consent of the Local Government Board, to proceed with the administrative extensions necessary for the complete scheme for enlarging Tooting Bec Mental Hospital. The proportion of the scheme which the managers at the present time propose to carry out is estimated to cost £88,000. Mr. T. W. Aldwinckle, F.R.I.B.A., has been appointed architect, subject to the approval of the Local Government Board.

### A UNIQUE RAILWAY ADVERTISEMENT.

A railway advertisement of unique character has recently been produced by the North Eastern Railway Company. It takes the form of a parody, in prose, verse, and picture, on Lewis Carroll's immortal heroine, "Alice in Wonderland," under the suggestive title of "Alice in Holidayland." Great pains and considerable skill have been exercised in the production of the booklet. On the cover appear the familiar figures such as the Walrus and Carpenter, Alice, the Mock Turtle, and the Gryphon, and inside are twelve excellent coloured plates illustrating the parody. The story cleverly introduces the advantages of holiday resorts, more especially for children, of such places as Scarborough, Whitby, Bridlington, Withernsea, and Filey, all of which can most conveniently be reached by the Company's line. A copy of the booklet can be obtained upon application to the passenger manager's office of the North Eastern Railway at York, or from station bookstalls.

### DOCTORS' WILLS.

MR. EDGAR JONES, F.R.C.S., of Great Burstead, Essex, who died on August 24, in his 103rd year, has left estate of which £12,458 is net personalty.

### Appointments.

Dr. I. A. Dowling has been elected an assistant medical officer to the out-patients' department of the Manchester Children's Hospital.

Dr. Ralph Smedley, of Shire Hall, Durham, has been appointed medical officer for West Sussex at a salary of £600 per annum, with travelling expenses.

Dr. F. Jefferson, Dr. J. H. Beverland, Dr. E. S. Dixon, and Dr. H. V. Walsh have been elected house surgeons at the Royal Victoria Hospital, Belfast.

Mr. Edward Mellanby, M.A., M.B. (Cantab.), late Beit Fellow, has been appointed Lecturer in Physiology in the department of Home Science and Economics at King's College for Women, London University.

Mr. G. Evans has resigned his appointment as second assistant medical officer at Bexley Mental Hospital. To fill the vacancy the third, fourth, and fifth assistant medical officers have each been promoted one grade.

Dr. C. Paget Lapage, M.R.C.P., one of the visiting physicians to the Manchester Children's Hospital, has been appointed Lecturer in Diseases of Children to the Victoria University of Manchester.

Mr. T. C. Clare, M.D., B.S. Lond., F.R.C.S. Eng., L.R.C.P. Lond., has been appointed honorary assistant surgeon at Leicester Royal Infirmary. His resident hospital experience includes the posts of resident surgeon and resident surgical officer, Birmingham Children's Hospital, one year; house surgeon, Queen's Hospital, Birmingham, one year; resident surgical officer, Chelsea Hospital, one year; and house surgeon, Gordon Hospital, one year.

### Personalia.

The Rev. S. E. Maughan-Ettrick has been appointed chaplain of Hanwell Mental Hospital.

Mr. Frank Cyril Tearks and Mr. Robert Nivison have been elected governors of Guy's Hospital.

Mr. William Buckley, J.P., chairman of the board of governors of the Manchester Children's Hospital, has been elected a member of the Flintshire County Council.

Mr. George Gregory, formerly head attendant at the Royal Edinburgh Mental Hospital, Craig House, Edinburgh, left personal estate of the value of £5,285.

Surgeon-General Arthur W. May, C.B., Director-General of the Medical Department of the Navy, has been granted the relative rank of Vice-Admiral in his Majesty's Fleet, from May 11.

Royal Army Medical Corps.—At the recent examination held on the termination of the Junior Course at the Royal Army Medical College, Lieut. C. J. H. Little was awarded the De Chaumont Prize and Lieut. L. Dunbar the Marshall Webb Prize. The Parkes Memorial Prize was awarded to Lieut. H. Beddingfield, and Lieut. F. C. Davidson received the second Montefiore and Tulloch Memorial Prizes.

In place of Mr. Francis J. A. Wood, resigned, Mr. G. Shrewsbury Smith has been elected chairman of Worcester General Infirmary. He was unanimously elected. For the office of vice-chairman, two gentlemen were nominated—Mr. Harris, as representing the city and Mr. R. Bruce Ward the county interest. When it was explained that the chairman would represent the city interest, Mr. Harris withdrew, and Mr. Bruce Ward was elected.

### Gifts and Bequests.

Miss Mary Bethia Savage, of West Kensington, has bequeathed £1,000 to St. George's Hospital for the endowment of a bed.

The After-Care Association for Blind, Deaf, and Cripple Children has received £100 from the Trustees of the City Parochial Charities.

Miss Helen Margaret Greswell, of Oxford, has bequeathed £1,000 to the Radcliffe Infirmary, Oxford, for the Endowment Fund.

Mr. Edmund Sweetman, of Co. Kildare, who left personal estate in the United Kingdom valued at £151,745, has bequeathed sums amounting to £1,150 to charities in Dublin and Kildare.

The Trustees of the late Sir Julius Wernher, Bart., have allocated £1,000 each to the Royal Hospital for Incurables, Putney Heath, and the British Home and Hospital for Incurables, Streatham, and £500 to St. Bartholomew's Hospital.

The Skinners' Company have made grants of £10 10s. each to the After-Care Association for Poor Persons Discharged and Recovered from Asylums for the Insane, Church House, Westminster, and the Royal Dental Hospital, Leicester Square.

The trusts under the will of Mrs. Margaret Pollock Glen, of Carlibar, Barrhead, contain the following provisions: To the Western Infirmary, Glasgow, the Royal Infirmary, Glasgow, the Victoria Infirmary, Glasgow, and the Royal Alexandra Infirmary, Paisley, £1,000 each.

Mr. Keddey Roy Fletcher, of Andover, has bequeathed, after various legacies, the residue of his estate on trust for his wife for life, with remainder as she may appoint, and in default of appointment then as to £1,000 to the London Hospital, for the endowment of a "Keddey Fletcher Bed."

The Trustees of the will of the late Sir Julius Wernher have allocated £300 to the Children's Sanatorium (for Phthisis), Holt, Norfolk; £250 to the After-Care Association for Blind, Deaf, and Cripple Children; £200 to the Royal Female Orphan Asylum, Beddington; and £1,000 to the London Orphan Asylum, Watford.

Mr. William Lees, of Sale, Cheshire, retired cotton spinner, has bequeathed £2,000 to the Oldham Infirmary, with the request that it should be used to endow two beds to be known as the "William Lees Bed" and the "Martha Lees Bed"; £1,000 each to the Oldham Deaf and Dumb Society, the Workshops for the Blind, Oldham, and the Manchester Hospital Sunday Fund for general purposes; and £1,500 among a number of charities mainly in Manchester.

### A TRIUMPH IN ICE-MAKING.

A very efficient hand-power ice-machine, known as the "W.K.," has been placed on the market by Messrs. Hannefords, Ltd., 91 and 93 Queen Victoria Street, E.C. The machine produces ice within two or three minutes from water of any temperature. The principle that water freezes when quickly evaporated is the basis of its construction, and this is carried into effect by means of a high-class piece of apparatus, in which the vacuum pump is the essential feature. Common commercial sulphuric acid is used as an absorber, but the design of the machine precludes the possibility of its contact with the water or other articles to be cooled. The apparatus combines simplicity of construction with perfect safety in use, and it should supply a much-felt want in small hospitals and similar institutions. The price, with all accessories, is £10, and the weight 50 lb.

